# Hofbauer Cells: Their Role in Healthy and Complicated Pregnancy

**DOI:** 10.3389/fimmu.2018.02628

**Published:** 2018-11-15

**Authors:** Leticia Reyes, Thaddeus G. Golos

**Affiliations:** ^1^Department of Pathobiological Sciences, School of Veterinary Medicine, University of Wisconsin-Madison, Madison, WI, United States; ^2^Department of Comparative Biosciences, Wisconsin National Primate Research Center, School of Veterinary Medicine, University of Wisconsin-Madison, Madison, WI, United States

**Keywords:** Hofbauer cells, placental macrophages, preeclampsia, villitis, chorioamnionitis, Zika, TORCH

## Abstract

Hofbauer cells are placental villous macrophages of fetal origin that are present throughout pregnancy. Although Hofbauer cell populations are antigenically and morphologically heterogeneous, their epigenetic, antigenic, and functional profiles most closely resemble alternatively activated macrophages or what are referred to as M2a, M2b, M2c, and M2d polarity subtypes. Consistent with an M2-like profile, these cells play an important role in placental development including vasculogenesis and angiogenesis. During placental inflammation Hofbauer cells may produce pro-inflammatory cytokines or mediators that damage the villous cell barrier, and induce fibrotic responses within the villi as a continuum of chronic inflammation. However, to date, there is no evidence that Hofbauer cells become classically activated or adopt an M1 polarity phenotype that is able to kill microbes. To the contrary, their predominant M2 like qualities may be why these cells are ineffective in controlling most TORCH infections. Moreover, Hofbauer cells may contribute to vertical transmission of various pathogens to the fetus since they can harbor live virus and serve as reservoirs within the placenta. The goal of this review is to summarize what is currently known about the role of Hofbauer cells in normal and complicated pregnancies that involve immunologic disorders, inflammation, and/or infection.

## Hofbauer cells in normal pregnancy

### Hofbauer cell location and proposed function

Hofbauer cells (HBC) originally referred to round to ovoid placental macrophages with a small nucleus and abundant vacuolated cytoplasm that could be identified by light microscopy (1). Subsequent ultrastructural studies further identified placental macrophages that were spindle or stellate shaped (2, 3) and that these cells are of fetal origin (4). HBC is now often used to describe any fetal derived placental macrophage that resides within the placental villous core, amnion, and chorionic lavae (5). HBCs are found in human placental tissue as early as 18 days post-conception (6) and remain throughout gestation (1).

HBCs are presumed to play a role in placental morphogenesis and homeostasis. HBCs are typically in apposition to endothelium and trophoblasts where they can mediate the function of these cells through paracrine signals or possibly cell-to-cell crosstalk (7–9). HBCs are pro-angiogenic in that they express large amounts of vascular endothelial growth factor (VEGF) (10, 11), and Sprouty (Spry) proteins Spry 1, 2, and 3 that modulate branching morphogenesis of placental villi (12). It has been suggested that HBC may participate in vasoregulation of placental blood vessels since they have the capacity to produce prostaglandin E_2_ and thromboxane *in vitro* (13).

### Phenotypic diversity of HBCs

**Figure 1:**
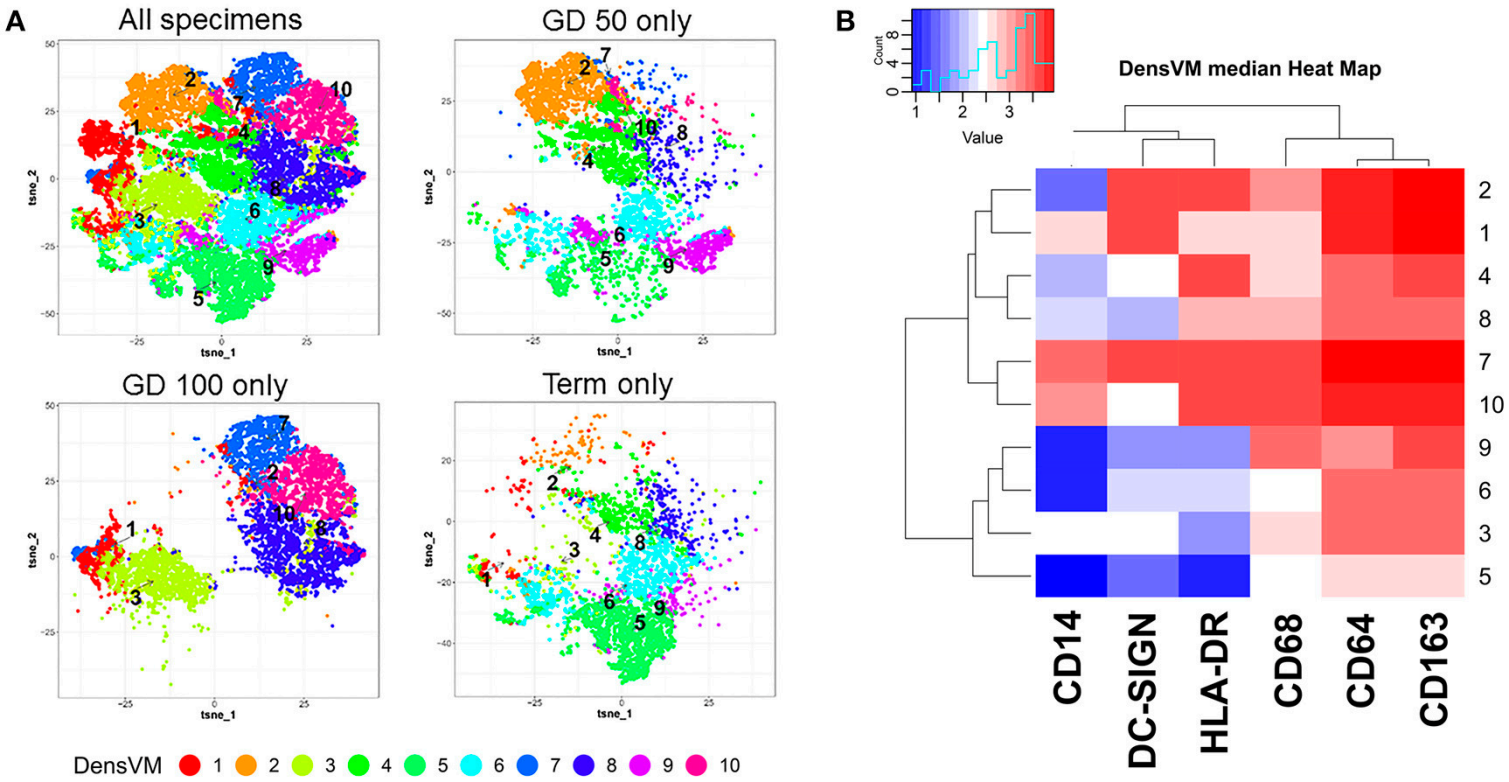
Marker defined HBC subsets within macaque placenta collected at different stages of gestation: Gestation day (GD) 50 ± 2 days (*n* = 2), GD100 ± 2 days (*n* = 2), and Term. **(A)** *t*-SNE visualization of DensVM generated HBC clusters of combined flow cytometry data from all gestation stages (All specimens) and by each individual gestation stage. **(B)** Marker and cluster specific DensVM median heatmap generated with flow cytometry data from all gestation stages. Clustering was ranked by both HBC cluster group (designated by row) and marker median expression (column). Images were created with Cytofkit ShinyAPP (22).

As is typical of macrophages, HBCs exhibit plasticity and their pleomorphism is likely a reflection of the complex and shifting microenvironment in which they reside (14–16). This has been verified through a variety of techniques including electron microscopy, immunohistochemistry, and flow cytometry. Initial descriptions of HBC pleomorphism came from ultrastructural observations that reported 4 types of macrophages based on their shape (3). Histochemical studies have further classified HBC subtypes by their expression of major histocompatibility complex (MHC) type II, complement receptors, lectins, lipopolysaccharide co-receptor (CD14), and CD68 that vary based on HBC location within the placenta as well as gestational stage (5, 15–19). Using multi-parameter flow cytometry coupled with serial gating of first trimester macaque HBCs, we previously identified two HBC subsets based on whether or not they expressed CD68 (14). To further illustrate this point we reanalyzed the dataset and added additional samples from late second trimester (gestation day 100), and near term (gestation day 160) (20, 21). We specifically measured the expression of HLA-DR, CD14, DC-SIGN, CD68, CD64, and CD163 in HBC by flow cytometry (Figure 1; Table 1, workflow detailed in Supplementary Material). Although our panel was not comprehensive, it included markers previously validated in rhesus macaque HBCs (18), some of which indicate innate immune activation, such as CD14 (23), or immune modulation (DC-SIGN, HLA-DR, CD68) (1, 15, 18, 24). In order to better capture the spectrum of HBC diversity, we used an unbiased approach to analyze the high-dimensional flow cytometric data. Raw flow cytometry data files were first processed with FlowJo, LLC version 10 software (Ashland, OR). Processed datasets from first (*n* = 2), second (*n* = 2), and third (*n* = 1) trimester pregnancies were then imported into Cytofkit (https://bioconductor.org/packages/cytofkit/), normalized, and analyzed with the DensVM computational clustering tool (22). *t*-Distributed Stochastic Neighbor Embedding (*t*-SNE) was used to created 2-dimensional maps of all HBC subsets generated by DensVM (Figure 1).

**Table 1:** Marker profiles of HBC subsets generated with DensVM clustering algorithm (22).

**Cluster**	**1st^a^**	**2nd^a^**	**3rd**	**Marker profile**
1	–	5 ± 4	3	CD163^Hi^/CD64^Hi^/CD68^Lo^/HLA-DR^+^/DC-SIGN^Hi^/CD14^Hi^
2	23 ± 22	–	3	CD163^Hi^/CD64^Hi^/CD68^Med^/HLA-DR^Hi^/DC-SIGN^Hi^/CD14^Lo^
3	–	24 ± 18	4	CD163^Med^/CD64^Med^/CD68^Lo^/HLA-DR^Lo^/DC-SIGN^Med^/CD14^Lo^
4	17 ± 1	–	10	CD163^Med^/CD64^Med^/CD68^Lo^/HLA-DR^Hi^/DC-SIGN^Med^/CD14^Lo^
5	8 ± 6	–	48	CD163^Lo^/CD64^Lo^/CD68^Lo^/HLA-DR^−^/DC-SIGN^Lo^/CD14^−^
6	11 ± 4	–	23	CD163^Med^/CD64^Med^/CD68^Lo^/HLA-DR^Med^/DC-SIGN^Med^/CD14^−^
7	2 ± 0.1	16 ± 5	–	CD163^Hi^/CD64^Hi^/CD68^Hi^/HLA-DR^Hi^/DC-SIGN^Hi^/CD14^Hi^
8	3 ± 1	24 ± 21	6	CD163^Med^/CD64^Med^/CD68^Med^/HLA-DR^Med^/DC-SIGN^Med^/CD14^Lo^
9	12 ± 3	–	3	CD163^Med^/CD64^Lo^/CD68^Hi^/HLA-DR^Lo^/DC-SIGN^Lo^/CD14^−^
10	2 ± 2	20 ± 2	–	CD163^+^/CD64^Hi^/CD68^Hi^/HLA-DR^Hi^/DC-SIGN^Med^/CD14^Hi^

a *Values represent the mean ± SD% positive cells per group (n = 2) per gestation group*.

With this approach we identified 10 HBC subsets within macaque placental tissues (Figure 1; Table 1). The 10 HBC subsets were subsequently validated by serial manual gating (Table 2). Both first and third trimester HBC populations were more diverse than the second trimester (8 vs. 5 clusters, respectively). This is not unusual given the physiological events that occur during these stages of pregnancy. For example, during the first trimester, HBCs are thought to participate in placental villous growth and tissue remodeling (13). At the same time HBCs in the vicinity of the placental bed may be affected by the inflammatory processes necessary for decidualization and embryo implantation (25). During the latter part of the third trimester, HBCs scattered through the placenta are exposed to various products released from senescent trophoblasts (26), necrotic cell debris associated with fibrinoid deposits within aging placental villi (13), and inflammatory mediators produced during parturition (27). These may promote the development of specialized subsets of HBCs in response to their microenvironment. Alternatively, they may represent different populations of fetal monocytes that are trafficking to the placenta across gestation.

**Table 2:** Marker profiles of HBC subsets generated by manual gating using FlowJo Software.

**Cluster**	**1st^a^**	**2nd^a^**	**3rd**	**Marker profile**
1	–	1.3 ± 0.4	8	CD163^Hi^/CD64^Hi^/CD68^Lo^/HLA-DR^+^/DC-SIGN^Hi^/CD14^Hi^
2	11 ± 11	–	1	CD163^Hi^/CD64^Hi^/CD68^Med^/HLA-DR^Hi^/DC-SIGN^Hi^/CD14^Lo^
3	–	20 ± 24	6	CD163^Med^/CD64^Med^/CD68^Lo^/HLA-DR^Lo^/DC-SIGN^Med^/CD14^Lo^
4	19 ± 4	–	3	CD163^Med^/CD64^Med^/CD68^Lo^/HLA-DR^Hi^/DC-SIGN^Med^/CD14^Lo^
5	8 ± 1	–	52	CD163^Lo^/CD64^Lo^/CD68^Lo^/HLA-DR^−^/DC-SIGN^Lo^/CD14^−^
6	9 ± 4	–	17	CD163^Med^/CD64^Med^/CD68^Lo^/HLA-DR^Med^/DC-SIGN^Med^/CD14^−^
7	1.8 ± 0.4	6 ± 3	–	CD163^Hi^/CD64^Hi^/CD68^Hi^/HLA-DR^Hi^/DC-SIGN^Hi^/CD14^Hi^
8	2.3 ± 1	17 ± 19	3.2	CD163^Med^/CD64^Med^/CD68^Med^/HLA-DR^Med^/DC-SIGN^Med^/CD14^Lo^
9	6 ± 4	–	2.5	CD163^Med^/CD64^Lo^/CD68^Hi^/HLA-DR^Lo^/DC-SIGN^Lo^/CD14^−^
10	0.5 ± 0.7	8 ± 7	–	CD163^+^/CD64^Hi^/CD68^Hi^/HLA-DR^Hi^/DC-SIGN^Med^/CD14^Hi^

a *Values represent the mean ± SD% positive cells per group (n = 2) per gestation group*.

**Figure 2:**
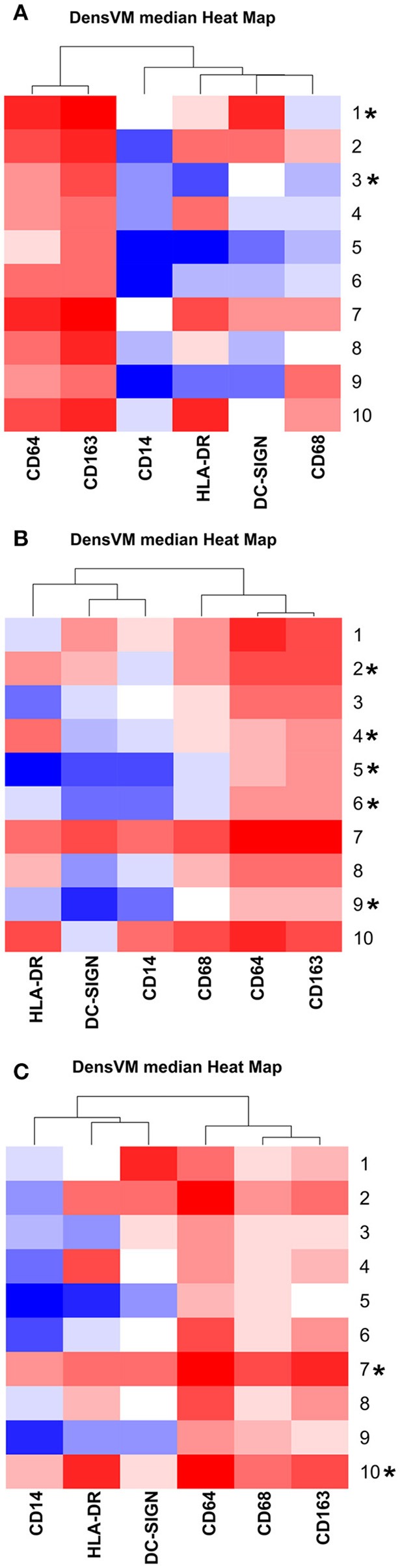
DensVM median heatmaps specific to each gestation stage. **(A)** GD50 ± 2 days, **(B)** GD100 ± 2 days (*n* = 2), and **(C)** Term (*n* = 1). Heatmaps were clustered by median expression of each marker (column). *Denotes HBC clusters that contained 1% of cells for that specific gestation stage. Images were created with Cytofkit ShinyAPP (22).

In order to assess gestation dependent changes in HBC subsets, marker expression heatmaps specific to each gestational stage were generated (Figure 2). We found that both CD163 and CD64 appeared to be constitutively expressed in all HBC subsets throughout pregnancy, indicating that these markers may be well-suited for the identification of HBCs in general. However, CD68 which is often used as a single marker to identify HBCs had variable expression over time. Namely, the intensity of CD68 expression peaked in the second trimester and significantly dropped as pregnancy progressed. This temporal expression pattern is similar to previous studies that described human HBC populations as changing in density as pregnancy progressed (1, 24).

As expected (5), there was at least one population of HBC that was negative for HLA-DR (cluster 5). Of the HBC subsets that were positive, HLA-DR expression followed a similar pattern to CD68 in that peak expression was observed in second trimester placenta. However, contrary to human studies (5), we found the proportion of HLA-DR positive cells in first trimester placenta to be greater than third trimester tissue. This may be due to timing of sampling since human studies evaluated tissues collected during 8–10 weeks gestation (5) whereas we examined tissues collected at a later gestational time point that developmentally would be equivalent to 17–18 weeks in human gestation.

DC-SIGN positive HBCs were present in all 3 stages of pregnancy; albeit with varying degrees of DC-SIGN expression. Consistent with Yang et al. (28) we found that DC-SIGN positive third trimester macaque HBCs co-expressed CD14, CD68, and CD163. In contrast to Bockle et al (29), we detected a large population of DC-SIGN positive HBC in third trimester placenta that co-expressed HLA-DR (23% in cluster 6). One caveat is that these cells had dim expression of both markers, which may explain why these cells may not be readily detected by immunofluorescent histology.

Of all the markers that we studied, the proportion of CD14 positive cells and their level of CD14 expression was the most diverse. Midgestation placenta, which is characterized by immune tolerance, had the greatest proportion of CD14^Hi^ positive HBCs that were also CD163^Hi^ (cluster 10). Although increased CD14 expression has been linked to pro-inflammatory HBCs (23), in normal pregnancy CD14 expression may represent an immune suppressed phenotype. CD14^Hi^/CD163^Hi^ expression as seen in cluster 10, is characteristic of immunosuppressive M2d or tumor associated macrophages (TAM) (30). CD14 positive HBCs also express anti-inflammatory TGF-β and IL-10 (31).

Even though this was a pilot experiment with a small sample size, our results demonstrate the potential of utilizing computational methods to analyze multidimensional data from HBCs. For instance, we obtained a global perspective on how HBC subsets change during pregnancy. Moreover, we gained insights into which macrophage markers may or may not be suitable for the general identification of HBCs by flow cytometric or immunohistochemical approaches.

### Functional diversity of HBCs

Mills et al. (32) were the first to introduce the concept that macrophage function could be polarized based on how the cell metabolized arginine. Arginine conversion to nitric oxide was linked to a macrophage that produced IFN-γ and inhibited wound healing, which was labeled as M1. Arginine conversion to ornithine was linked to a macrophage that produced TGF-β and promoted wound healing, this phenotype was labeled as M2. Over the years, the criteria for defining a macrophage as M1 has expanded (Table 2) to include microbicidal activity, and the production of pro-inflammatory cytokines and chemokines that promote cell mediated (TH1 type) responses. M2 polarity phenotype has been divided subsequently into M2a, M2b, M2c, and M2d subcategories (reviewed by Martinez et al. (33)), which are based on their responses to various agonists (Table 3). Collectively, M2 subtypes are linked by a dominant TH2 response profile, their development in response to fungal or helminth infections, and their role in tissue remodeling. However, M2b polarized macrophages share some qualities in M1 in that they can be pro-inflammatory by producing TNF, IL-1, and IL-6 along with IL-10, but they lack microbicidal activity.

**Table 3:** General features of macrophage polarity pertinent to HBC (30, 33, 34).

	**M1**	**M2a**	**M2b**	**M2c**	**M2d**
**Stimulus**	IFN-γ + LPS IFN-γ + TNF GM-CSF	IL-4 IL-13 Fungal and parasitic infections	Immune complexes IL-1R	IL-10 TGF-β Glucocorticoids	IL-6 LIF M-CSF Adenosine
**Surface markers**	CD80 MHC II	CD163 IL-1R MHC II CD206 IL-RN DC-SIGN	CD86 MHC II	CD163 TLR1 TLR8 CD206 CD14	CD163 CD14 CD85
**Secreted factors**	TNF IL-1β IL-6 IL-12 IL-23 CCL10 CCL11 CCL5 CCL8 CCL9 CCL2 CCL3 CCL4	IL-10 TGF-β IL-1ra	IL-1 IL-6 IL-10 TNF-α CCL1	IL-10 TGF-β CCR2 Pentraxin 3	VEGF MMP-9 IDO IL-10 IL-12 TNF-α TGF-β CCL5 CXCL10 CXCL16
**Biological effects**	TH1 responsesKilling intracellular pathogensTumor resistance	TH2 responsesKilling of extracellular responses	TH2 activationImmune regulation	Immune regulationMatrix deposition and tissue remodeling	Immune suppressionAngiogenesis

Since HBC are macrophages, it has been assumed that these cells protect the placenta and fetus from infection (13), which would be consistent with an M1 phenotype. However, there is no experimental evidence that HBCs within the placenta are capable of killing microbes (discussed in the next section of this review). Pro-M1 genes in HBC, such as TLR9, IL1B, IL12RB2, CD48, and FGR are silenced by methylation (35). On the other hand, pro-M2 genes, such as CCL2, CCL13, CCL14, CD209, and A2M are hypomethylated in HBCs and thus available for transcription (35). Collectively, HBCs isolated from term human placenta display M2a, M2b, and M2c characteristics based on cell surface marker profiles and cytokine expression (19, 31, 36). HBCs also share some features with M2d phenotype in that they are immune suppressive, pro-angiogenic, and co-express CD163 and CD14 (7, 10, 12, 36). Moreover, HBCs form multinucleated giant cells that express matrix metalloproteinase genes along with VEGF-C (37), which are also features of M2d. Hypothetically, a normal pregnancy has a balanced blend of HBC subtypes that functionally complement each other in providing optimal vascular development, villous growth and immune tolerance. Conversely, an imbalance in HBC subtypes may bring about or exacerbate pathologic pregnancy.

## The role of HBCs in pregnancy complications

### Villitis

Villitis is a histopathologic diagnosis with multiple underlying etiologies (38). It can be a consequence of hematogenous infection of the placenta by TORCH organisms that include Toxoplasma, Others (syphilis, varicella-zoster, parvovirus B19), Rubella, Cytomegalovirus (CMV), and Herpes infections (38, 39). However, most cases of villitis are not associated with infection, but may be immune mediated (40, 41). Acute villitis is usually caused by infection and it characterized by polymorphonuclear leukocytic infiltration of the villi with or without necrosis. *Listeria monocytogenes* infection during pregnancy is one of the most common causes of acute villitis (13, 42). Placental infection with *Treponema pallidin* (syphilis) may also present as acute villitis, but chronic villitis is more characteristic of congenital syphilis (43, 44). On the other hand chronic villitis is characterized by infiltration of the tissue by lymphocytes and macrophages. It may be accompanied by cellular proliferation and fibrosis of the villi. Most cases of villitis are multifocal and asymptomatic, but the lesion can be more extensive leading to preterm birth or miscarriage.

Perturbed HBC function is a common occurrence in chronic villitis. HBC hyperplasia or proliferation is seen in chronic villitis caused by infection with CMV, Zika, Herpes virus, Coxsackie, and villitis of unknown etiology (VUE) (38, 45–47). Regardless of the underlying cause, HBCs in chronic villitis exhibit an inflammatory phenotype. Satosar and colleagues showed an increase in the number of TNF positive HBCs with a concomitant decrease in SOCS-1 (suppressors of cytokine signaling) positive HBCs in villitis placentas positive for viral and bacterial infection (45). A similar response is also evident in VUE, which is now recognized to be an immune mediated process that resembles maternal anti-fetal rejection and placental graft vs. host disease (40). In VUE, hyperplastic HBCs are intermixed with infiltrating maternal macrophages and CD8^+^ T cells with an inflammatory transcriptome that is similar to the biological processes that occur during antigen presentation and immune response (40). In particular, HBCs in VUE are positive for CXCL9, CXCL10, CXCL11, and CXCL13 (40, 45). In this scenario HBCs are thought to be contributing to placental damage. In the case of TORCH infections, it is unknown whether HBCs are contributing to placental damage, or controlling infection. The presence of viral inclusions (CMV, Herpes Simplex, Coxsackie) or parasites (*Toxoplasma* and *Leishmania*) can certainly be found in HBCs, but it is unknown if these organisms are live and replication, or dying within the cell. HBCs are harboring live organisms these organisms are viable in these cells (38, 39).

HBC hyperplasia without villitis has also been observed with placental Zika virus infection (48, 49). This lesion is characterized by enlarged, hydropic chorionic villi, hyperplasia and focal proliferation of HBC, without necrosis or lymphocytic infiltration of the affected villi (48, 49). Some of the proliferating HBCs were found to contain Zika virus (49). Since Zika has been shown to replicate in HBCs (50–52), it has been proposed by several investigators that these cells may serve as a source of infection to the fetus (48, 53). However, the significance of HBCs in releasing infectious Zika virions is questionable since by Gavegnano and colleagues showed that Zika virions released from HBCs were incapable of infecting susceptible Vero cells (54). Whether antiviral responses in HBC are effective or compromised *in vivo* is yet to be determined. Regardless, perturbations in HBCs during placental infection with Zika suggest that these cells have altered function that may be detrimental to placental morphogenesis.

### Preterm delivery

There are a limited number of studies concerning HBCs in preterm delivery. These have been limited to chorioamnionitis-induced spontaneous preterm birth, severe preeclampsia, and HELLP syndrome. Two independent studies have shown that the density of CD68^+^ HBCs are significantly reduced in chorioamnionitis (24, 37). The underlying mechanism for a decrease in HBCs during chorioamnionitis is unknown but it has been speculated that these cells may be undergoing apoptosis (37). HBC function, particularly multinucleated giant cells, is altered in placentas with chorioamnionitis. Namely, these cells exhibited decreased tolerogenic activity compared to the same cells retrieved from normal pregnancies (37). The application of high dimensional flow cytometry may allow discernment of the significance of a decrease in the density of CD68^+^ cells: is CD68 selectively lost from a subset of cells? Does a different population of HBCs arise in these placentas?

Although a subset of preeclampsia patients develop HELLP, HELLP is considered a separate syndrome. Preeclampsia and HELLP have different clinical presentations (55, 56). Classical preeclampsia is characterized by hypertension and proteinuria, whereas HELLP involves activation of the coagulation system (55). Pathologic features within the placenta also differ between preeclampsia and HELLP. Infarction, intervillous thrombosis, and abruption is more common in placentas from preeclampsia patients than patients with HELLP (55). Furthermore, HBC numbers, and their expression of DC-SIGN and IL-10 are significantly reduced in patients with severe preeclampsia (28, 57). It has been suggested that the reduction of HBCs in preeclampsia may be promoting inflammatory damage due to the loss of tolerance-promoting HBCs (28). In contrast to preeclampsia, patients with HELLP exhibit increased numbers of CD68^+^ HBCs, and it was concluded that this may be due to increased inflammation or an adaptive response (56).

## Concluding remarks

Our understanding of the role of HBCs in pregnancy is still rudimentary, but current evidence provides a compelling argument that these cells are important in placental development and homeostasis. At least in some pregnancy complications, such as VUE and chorioamnionitis, HBC dysfunction may be contributing to disease pathogenesis. Since HBCs exhibit functional plasticity, they may be ideal targets for therapeutic manipulation during disease states. However, additional studies are needed to better define the functional role of various HBC subsets in both health and disease.

## Ethics statement

Macaque data presented in this article was obtained with approval from the University of Wisconsin Institutional Animal Care and Use Committee.

## Author contributions

LR conceived of the topic, conducted the research, and wrote the manuscript. TG assisted with non-human primate studies, and contributed to the writing of the manuscript.

### Conflict of interest statement

The authors declare that the research was conducted in the absence of any commercial or financial relationships that could be construed as a potential conflict of interest.
