# Supplementary material for: Hofbauer Cells: Their Role in Healthy and Complicated Pregnancy
Source: Front Immunol. 2018 Nov 15;9:2628. doi: 10.3389/fimmu.2018.02628 (PMC6249321; doi:10.3389/fimmu.2018.02628)
Supplement: Supplementary file 1 [file Data_Sheet_1.docx]

Supplementary Material

Hofbauer cells: their role in healthy and complicated pregnancy

Thaddeus Golo and Leticia Reyes*

*** Correspondence:** Leticia Reyes: lreyes2@wisc.edu

# Supplementary Methods

Isolation of macaque HBCs. All animal procedures were conducted under the approval of the University of Wisconsin – Madison Animal Care and Use Committee. Placental tissue was obtained by cesarean section and HBCs were isolated as previously described (57). However, placental cell suspensions were not enriched for HBCs by antibody based selection. Rather, cell suspensions were immediately stained for flow cytometry for markers summarized in Table S1 according to manufacturer’s instructions. Corresponding fluorescent minus 1 isotype controls were included with each experiment.

Macaque stained cell suspensions were analyzed with BD LSR Fortessa. The flow cytometer was calibrated with BDFACSDiva CS&T Research Beads prior to use. Compensation for each marker was determined with unstained cells, Ghost die, and antibody labeled UltraComp ebeads (Invitrogen). Flow cytometry data was exported as FCS files that were initially processed with FlowJo, LLC version 10 software (Ashland, OR). As shown in Figure S2, Dead cells were excluded prior to manual gating, which were set based on FMOs, then downsampled to 5000 counts. Downsampled FCS files from first (n =2), second (n =2) and third (n -1) trimester pregnancies were then imported into Cytofkit (https://bioconductor.org/packages/cytofkit/), normalized and merged by the ceil method prior to clustering with DensVM (22). t-Distributed Stochastic Neighbor Embedding (t-SNE) was used to create 2-dimensional maps of all HBC subsets generated by DensVM.

# Supplementary Figures and Tables

| Supplementary Table 1. Reagents used for macaques flow cytometry studies | | |
| --- | --- | --- |
| Marker | **Clone or tag** | **Manufacturer** |
| Dead | Ghost red 780 | Tonbo biosciences |
| CD163 | GHI/61 | BD Biosciences |
| CD64 | 10.1 | BD Biosciences |
| CD68 | Y1/82A | BD Biosciences |
| HLA-DR | G46-6 | BD Biosciences |
| DC-SIGN | DCN46 | BD Biosciences |
| CD14 | M5E2 | BD Biosciences |

## Supplementary Figures


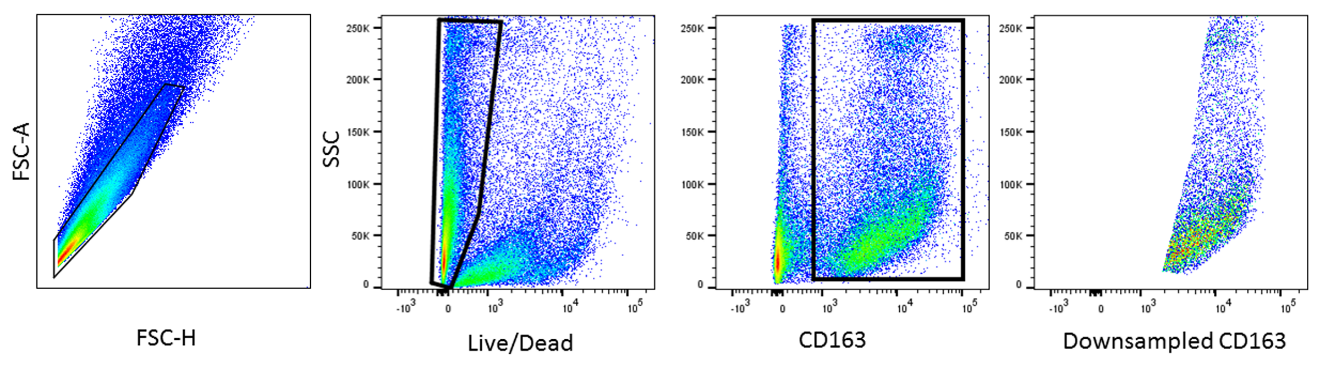


**Supplementary Figure 1.** Gating strategy for macaque HBCs.
